# Di-*n*-butyl­bis­[*N*-(2-meth­oxy­eth­yl)-*N*-methyl­dithio­carbamato-κ^2^ *S*,*S*′]tin(IV): crystal structure and Hirshfeld surface analysis

**DOI:** 10.1107/S2056989017001098

**Published:** 2017-01-27

**Authors:** Rapidah Mohamad, Normah Awang, Nurul F. Kamaludin, Mukesh M. Jotani, Edward R. T. Tiekink

**Affiliations:** a Biomedical Science Programme, School of Diagnostic and Applied Health Sciences, Faculty of Health Sciences, Universiti Kebangsaan Malaysia, Jalan Raja Muda Abdul Aziz, 50300 Kuala Lumpur, Malaysia; b Environmental Health and Industrial Safety Programme, School of Diagnostic and Applied Health Sciences, Faculty of Health Sciences, Universiti Kebangsaan Malaysia, Jalan Raja Muda Abdul Aziz, 50300 Kuala Lumpur, Malaysia; c Department of Physics, Bhavan’s Sheth R. A. College of Science, Ahmedabad, Gujarat 380 001, India; d Research Centre for Chemical Crystallography, School of Science and Technology, Sunway University, 47500 Bandar Sunway, Selangor Darul Ehsan, Malaysia

**Keywords:** crystal structure, organotin, di­thio­carbamate, Hirshfeld surface analysis

## Abstract

The complete mol­ecule of the title compound, [Sn(C_4_H_9_)_2_(C_5_H_10_NOS_2_)_2_], is generated by a crystallographic mirror plane, with the Sn^IV^ atom and the two inner methyl­ene C atoms of the butyl ligands lying on the mirror plane; statistical disorder is noted in the two terminal ethyl groups, which deviate from mirror symmetry. The di­thio­carbamate ligand coordinates to the metal atom in an asymmetric mode with the resulting C_2_S_4_ donor set defining a skew trapezoidal bipyramidal geometry; the *n*-butyl groups are disposed to lie over the longer Sn—S bonds. Supra­molecular chains aligned along the *a*-axis direction and sustained by methyl­ene-C—H⋯S(weakly coordinating) inter­actions feature in the mol­ecular packing. A Hirshfeld surface analysis reveals the dominance of H⋯H contacts in the crystal.

## Abstract 2

A skew trapezoidal bipyramidal coordination geometry based on a C_2_S_4_ donor set is found in the structure of (C_6_H_5_)_2_Sn[S_2_CN(Me)CH_2_CH_2_OMe]_2_, with the Sn^IV^ atom lying on a mirror plane.

## Chemical context   

The structural chemistry of mol­ecules with the general formula *R*_2_Sn(S_2_CN*RR*′)_2_ is diverse with coordination geometries ranging from five, as in trigonal bipyramid (*t*-Bu)_2_Sn(S_2_CNMe_2_)_2_ (Kim *et al.*, 1987 ▸), to seven, as in penta­gonal bipyramidal [MeOC(=O)CH_2_CH_2_]_2_Sn(S_2_CNMe)_2_ (Ng *et al.*, 1989 ▸). However, the overwhelming majority of structures are comprised of a six-coordinate Sn^IV^ atom, being based on either skew trapezoidal bipyramidal or octa­hedral coordination geometries (Tiekink, 2008 ▸). In the former, the di­thio­carbamate ligands are coord­in­ating in an asymmetric mode and lie in a plane, with the Sn-bound organic substituents orientated over the weaker Sn—S bonds. In the octa­hedral mol­ecules, the Sn-bound substituents occupy mutually *cis*-positions. As a general observation, compounds with Sn-bound aryl groups are octa­hedral and those with Sn-bound alkyl groups are skew trapezoidal bipyramidal. However, the capricious nature of the ultimate structure adopted in the solid state is nicely illustrated in a recent study whereby Ph_2_Sn[S_2_CN(CH_2_CH_2_OMe)Me]_2_, with a di­thio­carbamate ligand with dissimilar substituents, was found to be octa­hedral but, Ph_2_Sn[S_2_CN(CH_2_CH_2_OMe)_2_]_2_, with the di­thio­carbamate ligand having similar substituents, was skew trapezoidal bipyramidal (Mohamad, Awang, Jotani *et al.*, 2016 ▸). The structural inter­est notwithstanding, organotin di­thio­carbamates have potential biological applications, with recent investigations focusing upon biocidal activities, *e.g*. anti-fungal (Yu *et al.*, 2014 ▸) and anti-bacterial (Ferreira *et al.*, 2012 ▸), and, especially, as anti-cancer agents (Ferreira *et al.*, 2014 ▸; Kadu *et al.*, 2015 ▸), the focus of our inter­est (Khan *et al.*, 2014 ▸, 2015 ▸). During the course of the latter studies, crystals of the title compound, *n*-Bu_2_Sn[S_2_CN(CH_2_CH_2_OMe)Me]_2_, (I), became available. Herein, the crystal and mol­ecular structures of (I) are described along with a detailed analysis of the mol­ecular packing *via* an analysis of the Hirshfeld surface.
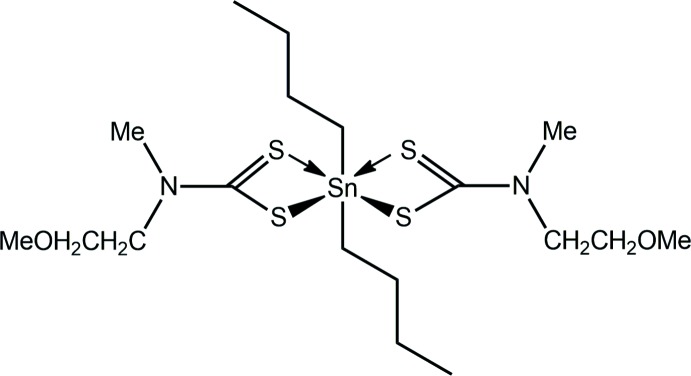


### Structural commentary   

**Figure 1:**
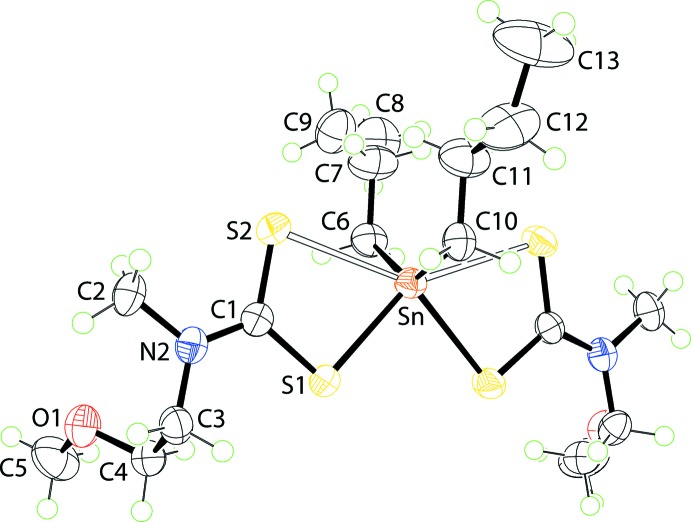
The mol­ecular structure of (I), showing the atom-labelling scheme and displacement ellipsoids at the 70% probability level. Unlabelled atoms are related by the symmetry operation (*x*, 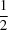 − *y*, *z*). Only one component of each of the disordered *n*-butyl groups is shown.

The asymmetric unit of (I) comprises half a mol­ecule being located on a crystallographic mirror plane with the Sn atom along with the two inner C atoms of the *n*-butyl groups lying on the plane, Fig. 1 ▸. The di­thio­carbamate ligand coordinates the Sn atom in an asymmetric fashion with the Δ(Sn—S), *i.e*. the difference between the Sn—S_long_ and Sn—S_short_ distances, being *ca* 0.39 Å, Table 1 ▸. This asymmetry is reflected in the associated C—S bond lengths with the short Sn—S bond being correlated with a long C—S bond length, Table 1 ▸. The coord­ination environment is completed by two α-C atoms of the *n*-butyl groups. The four S atoms are co-planar and define a skewed trapezoidal plane, and the α-C atoms are disposed over the weaker Sn—S bonds so that the C_2_S_4_ donor set defines a skew trapezoidal bipyramidal geometry.

## Supra­molecular features   

**Figure 2:**
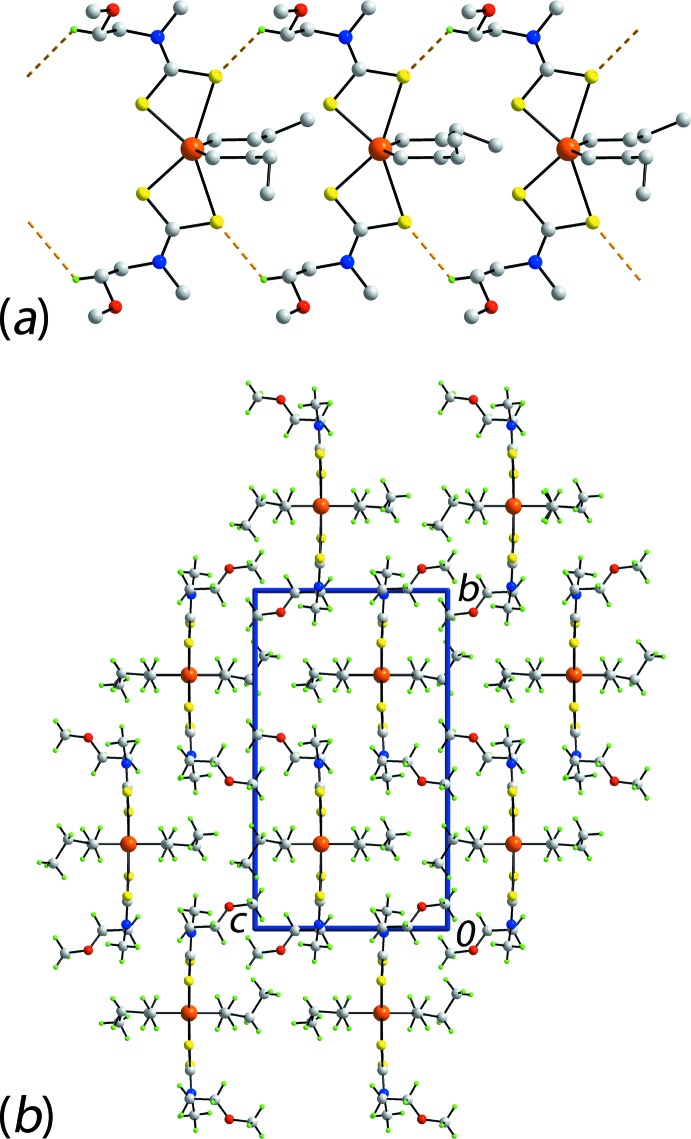
The mol­ecular packing in (I): (*a*) supra­molecular chain along the *a* axis sustained by methyl­ene-C—H⋯S inter­actions shown as orange dashed lines and (*b*) a view of the unit cell contents in projection down the *a* axis. Only one component of each of the disordered *n*-butyl groups is shown.

The only notable contacts identified in the mol­ecular packing are methyl­ene-C—H⋯S(weakly coordinating) inter­actions that assemble mol­ecules into linear supra­molecular chains propagating along the *a*-axis direction, Fig. 2 ▸ *a* and Table 2 ▸. The chains pack in the crystal with no specific inter­actions between them, Fig. 2 ▸ *b*. In order to ascertain more information of the nature of inter­actions between mol­ecules, the mol­ecular packing and its Hirshfeld surface was analysed, as discussed in *Hirshfeld surface analysis*.

## Hirshfeld surface analysis   

**Figure 3:**
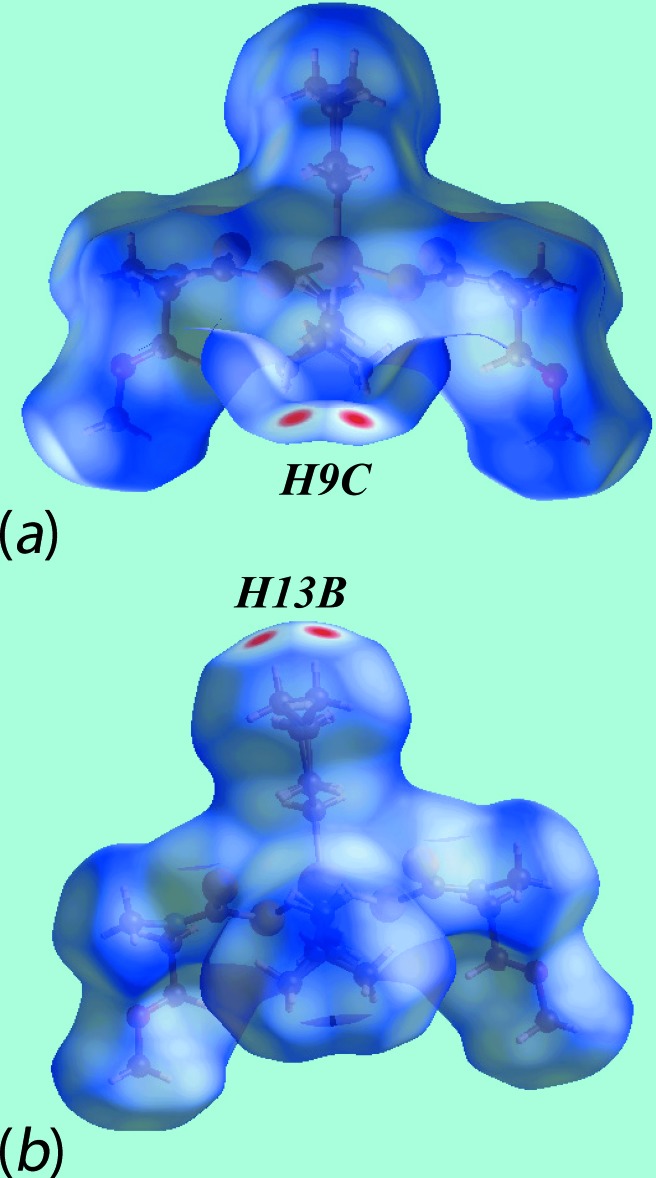
Two views of the Hirshfeld surface mapped over *d*_norm_ for (I). The disorder component has been retained in the images.

**Figure 4:**
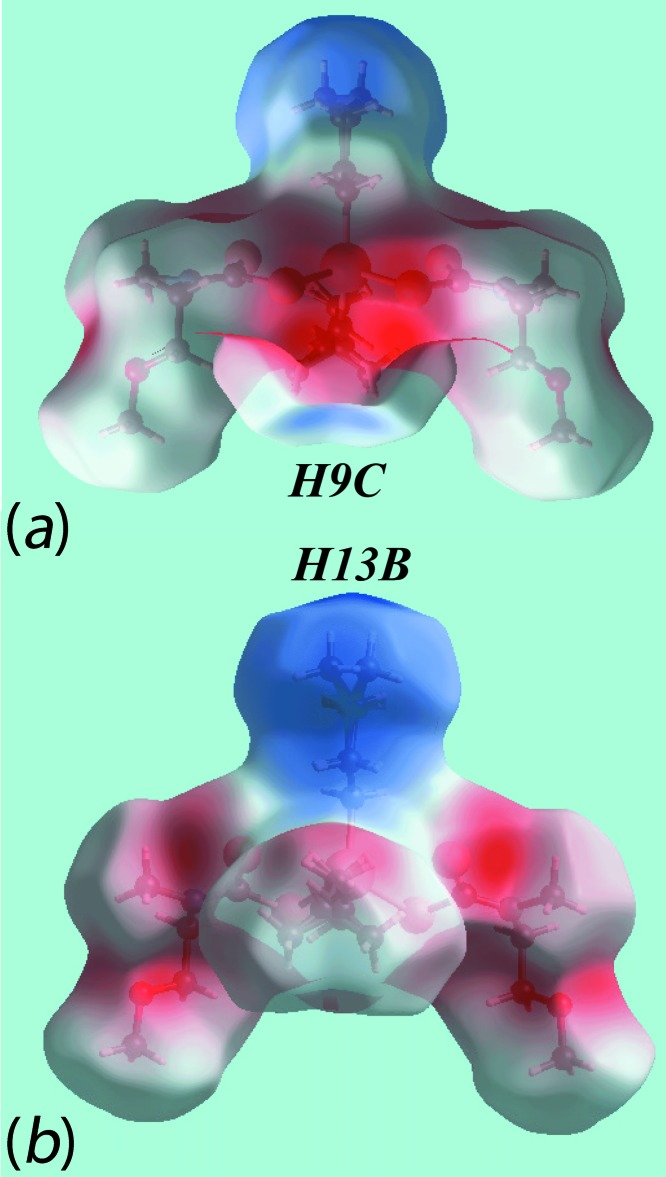
Two views of the Hirshfeld surfaces mapped over the electrostatic potential highlighting the disparate charge about the terminal hydrogen atoms (the red and blue regions represent negative and positive electrostatic potentials, respectively) for (I).

**Figure 5:**
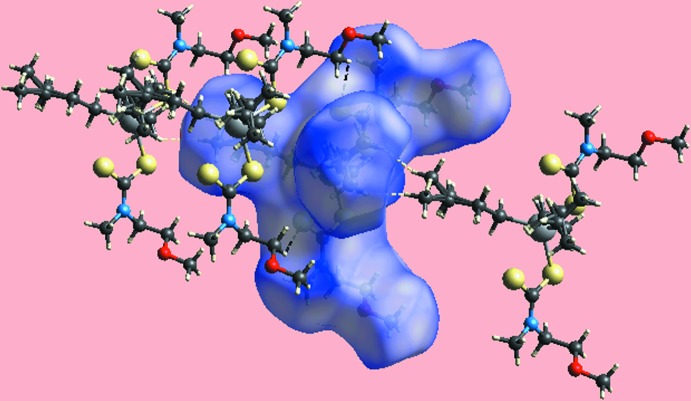
A view of the Hirshfeld surface mapped over *d*_norm_ for a reference mol­ecule in contact with nearest neighbouring mol­ecules and highlighting inter­molecular C—H⋯S and H⋯H inter­actions, shown as white and black dashed lines, respectively.

The Hirshfeld surface analysis for (I) was performed as described recently for organotin di­thio­carbamates (Mohamad, Awang, Kamaludin *et al.*, 2016 ▸). From the views of the Hirshfeld surface mapped over *d*_norm_, in the range −0.298 to +1.346 au, in Fig. 3 ▸, the pairs of bright-red spots near hydrogen atoms H9*C* and H13*B* of the disordered methyl groups, *i.e*. deviating from mirror symmetry, indicate their participation in specific inter­molecular H⋯H inter­actions. In the crystal, these lead to a supra­molecular chain along the *c* axis. The presence of this di­hydrogen inter­action, resulting from disparate charges on respective hydrogen atoms, can also be viewed by the different curvatures and electrostatic potentials around these atoms on the Hirshfeld surface mapped over the electrostatic potential in the range −0.082 to +0.163 au, Fig. 4 ▸. Fig. 5 ▸ illustrates the immediate environment around a reference mol­ecule within its Hirshfeld surface mapped over *d*_norm_, highlighting the inter­molecular C—H⋯S and H⋯H inter­actions.

**Figure 6:**
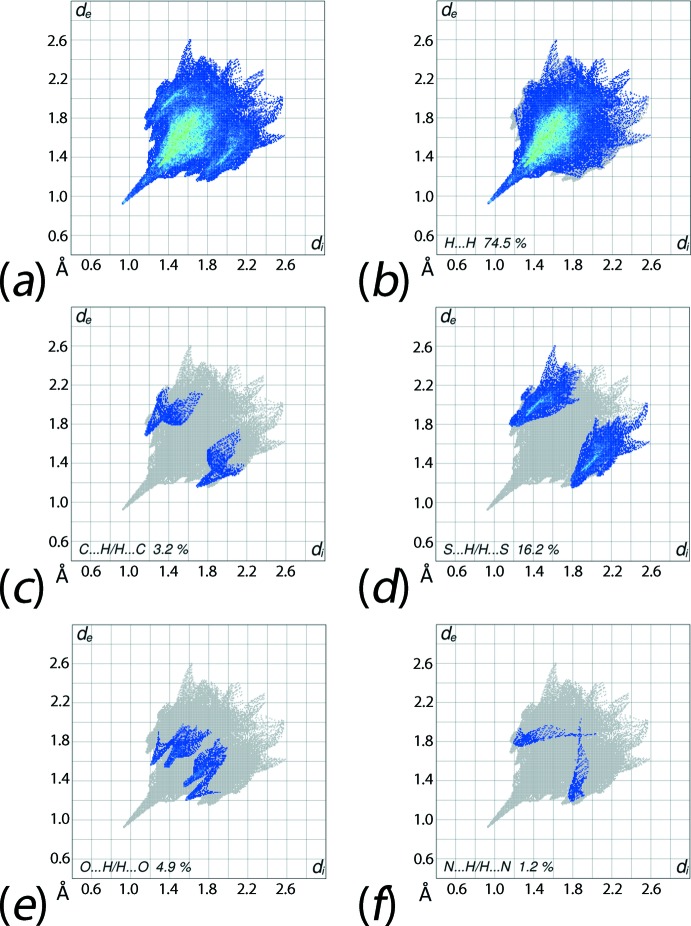
Views of the (*a*) full two-dimensional fingerprint plot for (I), and plots delineated into (*b*) H⋯H, (*c*) C⋯H/H⋯C, (*d*) S⋯H/H⋯S, (*e*) O⋯H/H⋯O and (*f*) N⋯H/H⋯N contacts.

From the overall two dimensional fingerprint plot, Fig. 6 ▸ *a*, and those delineated (McKinnon *et al.*, 2007 ▸) into H⋯H, C⋯H/H⋯C, S⋯H/H⋯S, O⋯H/H⋯O and N⋯H/H⋯N contacts, illustrated in Fig. 6 ▸ *b*–*f*, it is inter­esting to note that each of the specified inter­atomic contacts involves the participation of H atoms to the Hirshfeld surfaces. The qu­anti­tative summary showing the relative contributions from all inter­atomic contacts, given in Table 3 ▸, reinforces this fact.

In the fingerprint plot delineated into H⋯H contacts, Fig. 6 ▸ *b*, a long and distinctive spike at *d*_e_ + *d*_i_ ∼ 1.8 Å represents H⋯H bonding described above, Table 4 ▸, *i.e*. between methyl-H9*B* and -H13*B* atoms. The major contribution from these contacts to the Hirshfeld surface, *i.e*. 74.5%, and the essentially same shape of overall and H⋯H delineated fingerprint plots in the upper (*d*_e_, *d*_i_) region, Fig. 6 ▸ *a* and *b*, show the dominance of these inter­actions in the mol­ecular packing. The peak in the plot corresponding to a second short inter­atomic H⋯H contact, *i.e*. between methyl-H2*B* and methyl­ene-H10*A*, Table 4 ▸, is diminished within the plot due to H9*B*⋯H13*B* inter­action. The di­hydrogen H⋯H bonding also results in short inter­atomic C⋯H/H⋯C contacts, Table 4 ▸, leading to a pair of short peaks at *d*_e_ + *d*_i_ ∼ 2.8 Å in the delineated fingerprint plot, Fig. 6 ▸ *c*; the other inter­atomic short C⋯H/H⋯C contact is merged within the plot. The presence of the weak C—H⋯S inter­actions, Table 2 ▸, is seen from the fingerprint plot corresponding to S⋯H/H⋯S contacts, Fig. 6 ▸ *d*, and is evident as a pair of broad peaks at *d*_e_ + *d*_i_ ∼ 2.9 Å. The fingerprint plots delineated into O⋯H/H⋯O and N⋯H/H⋯N contacts, Fig. 6 ▸ *e* and *f*, contribute in a minor fashion to the Hirshfeld surface and their characteristic points are longer than their respective van der Waals separations, *i.e*. longer than 2.72 and 2.75 Å, respectively, and hence it is likely they do not make any significant contribution to the mol­ecular packing.

A comment on the relationship of the modelled disorder, the contribution of H⋯H contacts to the Hirshfeld surface and the nature of the H⋯H contacts is warranted. In the statistical disorder model for (I), it might be normally assumed (as done in Fig. 2 ▸ *b*) that that H atoms adopt positions as far apart from each other as possible rather than participate in ‘non-bonded steric repulsion’ (Matta *et al.*, 2003 ▸). In (I), this does not appear to the case but, rather is an example where H⋯H contacts contribute to the stabilization of the mol­ecular packing. In examples where di­hydrogen H⋯H contacts are formed intra­molecularly, energies of stabilization up to 10 kcal mol^−1^ have been suggested (Matta *et al.*, 2003 ▸).

## Database survey   

The inter­est in organotin di­thio­carbamates is reflected in the relatively large number of crystal structures available in the crystallographic literature (Groom *et al.*, 2016 ▸). An example of this inter­est is twenty structures conforming to the general formula *n*-Bu_2_Sn(S_2_CN*RR*’)_2_. One structure, *i.e. R* = *R*′ = *i*-Pr (Farina *et al.*, 2000 ▸), conforms to crystallographic *mm*2 symmetry (implying disorder in the terminal residues), seven, *i.e. R* = Me, *R*′ = *n*-Bu (Ramasamy *et al.*, 2013 ▸), *R* = Me, *R*′ = CH_2_C(H)Me_2_ (Ferreira *et al.*, 2012 ▸), *R* = Me, *R*′ = methyl­ene-1,3-dioxolan-2-yl (Ferreira *et al.*, 2012 ▸), *R* = Et, *R*′ = methyl­ene-4-pyridyl (Barba *et al.*, 2012 ▸), N*R*,*R*′ = piperidine (Khan *et al.*, 2015 ▸), N*RR*′ = morpholine (Vrábel & Kellö, 1993 ▸) and N*RR*′ = 4-(2-meth­oxy­phen­yl)piperazine (Zia-ur-Rehman *et al.*, 2012 ▸), have twofold symmetry with the remainder having no crystallographically imposed symmetry. This implies the structure of (I) is the first of this type to have crystallographic *m* symmetry. Two structures, *i.e. R* = *R*′ = Et (Vrábel *et al.*, 1992 ▸) and *R* = *R*′ = *n*-Bu (Ramasamy *et al.*, 2013 ▸), have two independent mol­ecules in the crystallographic unit and, remarkably, one, *i.e. R* = *i*-Pr and *R*′ = benzyl (Awang, Baba, Yousof *et al.*, 2010 ▸), has Z′ = 5. In all, there are 26 independent di­thio­carbamate ligands in *n*-Bu_2_Sn(S_2_CN*RR*′)_2_.

The first noteworthy comment to be made on the structures of *n*-Bu_2_Sn(S_2_CN*RR*′)_2_ is that they all conform to the same structural motif as adopted for (I). The Sn—S_short_ bond lengths in these structures span a relatively narrow range of 2.51 to 2.55 Å and cluster around 2.53 Å. As might be anti­cipated, a wider range is exhibited by the Sn—S_long_ bonds, *i.e*. 2.83 to 3.08 Å and these cluster around 2.96 Å. Given the range of Sn—S_short_ bond lengths is 0.04 Å and that for Sn—S_long_ is 0.25 Å, the observation that differences between the average values of Sn—S_short_ and Sn—S_long_ span a range of 0.43 Å indicates no specific correlations exist between Sn—S_short_ and Sn—S_long_ bond lengths. The S_short_—Sn—S_short_, S_long_—Sn—S_long_ and C—Sn—C angles cluster around 83, 147 and 136°, respectively. However, these angles span ranges of 8° (range: 80 to 88°), 10° (140 to 151°) and 18° (127 to 145°), respectively. The disparity in the S—Sn—S angles is as expected from the adopted coordination geometry. While, generally, the S_long_—Sn—S_long_ angles are wider than the C—Sn—C angles, there are three exceptional structures, namely *R* = *R*′ = Et (Vrábel *et al.*, 1992 ▸), *R* = Et and *R*′ = Cy (Awang, Baba, Yamin *et al.*, 2010 ▸) and *R* = benzyl and *R*′ = methyl­ene-4-pyridyl (Gupta *et al.*, 2015 ▸) have C—Sn—C which are marginally wider, by *ca* 1°, than the S_long_—Sn—S_long_ angles. The fact of non-systematic variations in the geometric parameters in organotin di­thio­carbamates has been commented upon previously (Buntine *et al.*, 1998 ▸; Muthalib *et al.*, 2014 ▸).

The homogeneity in the *n*-Bu_2_Sn(S_2_CN*RR*′)_2_ structural motif does not translate to the diphenyl analogues, *i.e*. Ph_2_Sn(S_2_CN*RR*′)_2_. Of the 19 structures conforming to this general formula, seven resemble the skew trapezoidal bipyramidal motif with the majority, *i.e*. twelve, having a *cis*-disposition of the tin-bound phenyl substituents. In this context, it is noteworthy that all structures of the general formula Sn(S_2_CN*RR*′)_2_ *X*_2_, where *X* = halide, are invariably *cis*-S_4_ *X*_2_ octa­hedral (Tiekink, 2008 ▸). Given the electronegativity of a phenyl group is inter­mediate between that of an alkyl group and a halide, it seems that there is a fine balance between adopting one structural motif over the other for Ph_2_Sn(S_2_CN*RR*′)_2_ compounds.

## Synthesis and crystallization   

(2-Meth­oxy­eth­yl)methyl­amine (10 mmol) dissolved in ethanol (30 ml) was stirred in an ice bath (*ca* 277 K) for 30 min. 25% Ammonia solution (*ca* 2 ml) was added to make the solution basic. Then, a cold ethanol solution of carbon di­sulfide (10 mmol) was added to the solution followed by stirring for about 2 h. Next, di-*n*-butyl­tin(IV) dichloride (5 mmol), dissolved in ethanol (30 ml), was added to the solution which was further stirred for 2 h. The precipitate that formed was filtered and then washed three times with cold ethanol to remove any impurities. The precipitate was then dried in a dessicator. The compound was crystallized in a mixture of chloro­form and ethanol (1:2 *v*/*v*) at room temperature to give colourless slabs. Yield: 66%, m.p. 333–336 K. Analysis. Found C, 40.3; H, 7.3; N, 5.0; S, 22.8. C_18_H_38_N_2_O_2_S_4_Sn requires: C, 38.5; H, 6.8; N, 5.0; S, 23.7. IR (cm^−1^): 1490 ν(C—N), 991 ν(C—S), 553 ν(Sn—C), 420 ν(Sn—S). ^1^H NMR (CDCl_3_): 7.40–7.74 (15H, Sn–Ph), 4.07 (2H, OCH_2_), 3.71 (2H, NCH_2_), 3.46 (3H, OCH_3_), 3.40 (3H, NCH_3_), 2.04 (2H, SnCH_2_), 1.92 (2H, SNCH_2_C*H*_2_), 1.44 (2H, C*H*_2_CH_3_), 0.98 (3H, CH_2_C*H*_3_). ^13^C{^1^H} NMR (CDCl_3_): δ 201.2 (S_2_C), 70.1 (OCH_2_), 59.1 (NCH_2_), 56.6 (OCH_3_), 44.5 (NCH_3_), 34.3 (SnCH_2_), 28.6 (SnCH_2_ *C*H_2_), 26.5 (*C*H_2_CH_3_), 13.9 (CH_2_ *C*H_3_). ^119^Sn{^1^H} NMR (CDCl_3_): 338.6.

## Refinement   

Crystal data, data collection and structure refinement details are summarized in Table 5 ▸. Carbon-bound H atoms were placed in calculated positions (C—H = 0.98–0.99 Å) and were included in the refinement in the riding model approximation, with *U*_iso_(H) set to 1.2–1.5*U*_eq_(C). The mol­ecule has crystallographic mirror symmetry with the Sn atom and *n*-butyl-C atoms lying on the plane. The terminal CH_2_CH_3_ residue of each *n*-butyl group is statistically disordered across this plane. Owing to poor agreement, three reflections, *i.e*. (172), (124) and (155), were omitted from the final cycles of refinement.

## Supplementary Material

Crystal structure: contains datablock(s) I, global. DOI: 10.1107/S2056989017001098/hb7654sup1.cif

Structure factors: contains datablock(s) I. DOI: 10.1107/S2056989017001098/hb7654Isup2.hkl

CCDC reference: 1528948

Additional supporting information:  crystallographic information; 3D view; checkCIF report

## Figures and Tables

**Table 1:** Selected bond lengths (Å)

Sn—S1	2.5425 (5)	Sn—C10	2.138 (3)
Sn—S2	2.9318 (5)	S1—C1	1.7443 (18)
Sn—C6	2.146 (3)	S2—C1	1.6974 (19)

**Table 2:** Hydrogen-bond geometry (Å, °)

*D*—H⋯*A*	*D*—H	H⋯*A*	*D*⋯*A*	*D*—H⋯*A*
C4—H4*B*⋯S2^i^	0.99	2.96	3.608 (2)	124

Symmetry code: (i) 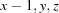.

**Table 3:** Percentage contribution of the different inter­molecular contacts to the Hirshfeld surface in (I)

Contact	% contribution in (I)
H⋯H	74.5
S⋯H/H⋯S	16.2
O⋯H/H⋯O	4.9
C⋯H/H⋯C	3.2
N⋯H/H⋯N	1.2

**Table 4:** Short inter­atomic contacts in (I)

Contact	distance	symmetry operation
H9*C*⋯H13*B*	1.85	*x*, *y*, 1 + *z*
H2*B*⋯H10*A*	2.27	1 − *x*, −*y*, 1 − *z*
C9⋯H13*B*	2.72	*x*, *y*, 1 + *z*
C13⋯H9*C*	2.73	*x*, *y*, −1 + *z*
C1⋯H2*A*	2.86	1 − *x*, −*y*, 1 − *z*
S2⋯H4*B*	2.96	1 + *x*, *y*, *z*

**Table 5:** Experimental details

Crystal data	
Chemical formula	[Sn(C_4_H_9_)_2_(C_5_H_10_NOS_2_)_2_]
*M*_r_	561.43
Crystal system, space group	Monoclinic, *P*2_1_/*m*
Temperature (K)	148
*a*, *b*, *c* (Å)	7.1021 (4), 18.0761 (8), 10.8809 (7)
β (°)	108.877 (7)
*V* (Å^3^)	1321.74 (14)
*Z*	2
Radiation type	Mo *K*α
μ (mm^−1^)	1.30
Crystal size (mm)	0.50 × 0.42 × 0.40

Data collection	
Diffractometer	Agilent Technologies SuperNova Dual diffractometer with an Atlas detector
Absorption correction	Multi-scan (*CrysAlis PRO*; Agilent, 2015 ▸)
*T*_min_, *T*_max_	0.482, 1.000
No. of measured, independent and observed [*I* > 2σ(*I*)] reflections	10631, 4063, 3712
*R*_int_	0.022
(sin θ/λ)_max_ (Å^−1^)	0.739

Refinement	
*R*[*F* ^2^ > 2σ(*F* ^2^)], *wR*(*F* ^2^), *S*	0.027, 0.072, 1.12
No. of reflections	4063
No. of parameters	147
No. of restraints	2
H-atom treatment	H-atom parameters constrained
Δρ_max_, Δρ_min_ (e Å^−3^)	0.68, −0.56

Computer programs: *CrysAlis PRO* (Agilent, 2015 ▸), *SHELXL97* (Sheldrick, 2008 ▸), *SHELXL2014* (Sheldrick, 2015 ▸), *ORTEP-3 for Windows* (Farrugia, 2012 ▸), *DIAMOND* (Brandenburg, 2006 ▸) and *publCIF* (Westrip, 2010 ▸).

## supplementary crystallographic information

### Crystal data

**Table:**

[Sn(C_4_H_9_)_2_(C_5_H_10_NOS_2_)_2_]	*F*(000) = 580
*M**_r_* = 561.43	*D*_x_ = 1.411 Mg m^−^^3^
Monoclinic, *P*2_1_/*m*	Mo *K*α radiation, λ = 0.71073 Å
*a* = 7.1021 (4) Å	Cell parameters from 6472 reflections
*b* = 18.0761 (8) Å	θ = 4.5–31.4°
*c* = 10.8809 (7) Å	µ = 1.30 mm^−^^1^
β = 108.877 (7)°	*T* = 148 K
*V* = 1321.74 (14) Å^3^	Block, colourless
*Z* = 2	0.50 × 0.42 × 0.40 mm

### Data collection

**Table:**

Agilent Technologies SuperNova Dual diffractometer with an Atlas detector	4063 independent reflections
Radiation source: SuperNova (Mo) X-ray Source	3712 reflections with *I* > 2σ(*I*)
Mirror monochromator	*R*_int_ = 0.022
Detector resolution: 10.4041 pixels mm^-1^	θ_max_ = 31.7°, θ_min_ = 3.8°
ω scan	*h* = −6→10
Absorption correction: multi-scan (CrysAlis PRO; Agilent, 2015)	*k* = −26→25
*T*_min_ = 0.482, *T*_max_ = 1.000	*l* = −15→12
10631 measured reflections	

### Refinement

**Table:**

Refinement on *F*^2^	2 restraints
Least-squares matrix: full	Hydrogen site location: inferred from neighbouring sites
*R*[*F*^2^ > 2σ(*F*^2^)] = 0.027	H-atom parameters constrained
*wR*(*F*^2^) = 0.072	*w* = 1/[σ^2^(*F*_o_^2^) + (0.0308*P*)^2^ + 0.5383*P*] where *P* = (*F*_o_^2^ + 2*F*_c_^2^)/3
*S* = 1.12	(Δ/σ)_max_ = 0.002
4063 reflections	Δρ_max_ = 0.68 e Å^−^^3^
147 parameters	Δρ_min_ = −0.56 e Å^−^^3^

### Special details

**Table:**

Geometry. All esds (except the esd in the dihedral angle between two l.s. planes) are estimated using the full covariance matrix. The cell esds are taken into account individually in the estimation of esds in distances, angles and torsion angles; correlations between esds in cell parameters are only used when they are defined by crystal symmetry. An approximate (isotropic) treatment of cell esds is used for estimating esds involving l.s. planes.

### Fractional atomic coordinates and isotropic or equivalent isotropic displacement parameters (Å^2^)

**Table:**

	*x*	*y*	*z*	*U*_iso_*/*U*_eq_	Occ. (<1)
Sn	0.63209 (3)	0.2500	0.65708 (2)	0.02489 (6)	
S1	0.36767 (7)	0.15500 (2)	0.65625 (5)	0.02861 (11)	
S2	0.76229 (7)	0.09574 (3)	0.66305 (5)	0.02966 (11)	
O1	0.3352 (2)	−0.06447 (9)	0.86623 (16)	0.0396 (3)	
N1	0.4586 (2)	0.01192 (8)	0.66892 (16)	0.0266 (3)	
C1	0.5266 (3)	0.08039 (10)	0.66318 (17)	0.0234 (3)	
C2	0.5918 (3)	−0.05193 (11)	0.6850 (2)	0.0341 (4)	
H2A	0.6388	−0.0555	0.6099	0.051*	
H2B	0.5197	−0.0972	0.6916	0.051*	
H2C	0.7059	−0.0458	0.7642	0.051*	
C3	0.2511 (3)	−0.00359 (11)	0.6614 (2)	0.0298 (4)	
H3A	0.2103	−0.0516	0.6170	0.036*	
H3B	0.1637	0.0351	0.6080	0.036*	
C4	0.2207 (3)	−0.00634 (11)	0.7922 (2)	0.0325 (4)	
H4A	0.2615	0.0413	0.8380	0.039*	
H4B	0.0781	−0.0144	0.7807	0.039*	
C5	0.2984 (4)	−0.07280 (17)	0.9863 (3)	0.0531 (7)	
H5A	0.3355	−0.0272	1.0371	0.080*	
H5B	0.3776	−0.1141	1.0350	0.080*	
H5C	0.1568	−0.0829	0.9697	0.080*	
C6	0.8488 (4)	0.2500	0.8477 (3)	0.0309 (6)	
H6A	0.8300	0.2943	0.8959	0.037*	0.5
H6B	0.8300	0.2057	0.8959	0.037*	0.5
C7	1.0587 (5)	0.2500	0.8393 (3)	0.0453 (8)	
H7A	1.0830	0.2982	0.8039	0.054*	0.5
H7B	1.0677	0.2111	0.7773	0.054*	0.5
C8	1.2246 (7)	0.2366 (3)	0.9707 (5)	0.0471 (18)	0.5
H8A	1.1858	0.1960	1.0186	0.056*	0.5
H8B	1.3502	0.2226	0.9557	0.056*	0.5
C9	1.2526 (10)	0.3068 (4)	1.0476 (7)	0.0674 (16)*	0.5
H9A	1.2572	0.3488	0.9916	0.101*	0.5
H9B	1.3776	0.3043	1.1202	0.101*	0.5
H9C	1.1413	0.3133	1.0813	0.101*	0.5
C10	0.6376 (4)	0.2500	0.4618 (3)	0.0278 (5)	
H10A	0.5652	0.2058	0.4167	0.033*	0.5
H10B	0.5652	0.2942	0.4167	0.033*	0.5
C11	0.8436 (5)	0.2500	0.4497 (3)	0.0442 (8)	
H11A	0.9132	0.2039	0.4883	0.053*	0.5
H11B	0.9200	0.2923	0.4994	0.053*	0.5
C12	0.8384 (7)	0.2556 (15)	0.3070 (4)	0.059 (3)	0.5
H12A	0.7967	0.2074	0.2637	0.071*	0.5
H12B	0.7378	0.2929	0.2615	0.071*	0.5
C13	1.0384 (9)	0.2771 (5)	0.2943 (6)	0.086 (3)	0.5
H13A	1.0801	0.3251	0.3361	0.129*	0.5
H13B	1.0263	0.2805	0.2021	0.129*	0.5
H13C	1.1378	0.2395	0.3364	0.129*	0.5

### Atomic displacement parameters (Å^2^)

**Table:**

	*U*^11^	*U*^22^	*U*^33^	*U*^12^	*U*^13^	*U*^23^
Sn	0.02457 (10)	0.02545 (9)	0.02660 (10)	0.000	0.01098 (7)	0.000
S1	0.0263 (2)	0.0217 (2)	0.0407 (3)	0.00326 (16)	0.0147 (2)	0.00337 (18)
S2	0.0279 (2)	0.0278 (2)	0.0364 (3)	0.00582 (17)	0.0146 (2)	0.00320 (18)
O1	0.0414 (8)	0.0371 (8)	0.0389 (9)	0.0048 (6)	0.0112 (7)	0.0136 (7)
N1	0.0304 (8)	0.0225 (7)	0.0267 (8)	0.0017 (6)	0.0091 (7)	−0.0005 (6)
C1	0.0269 (8)	0.0243 (8)	0.0194 (8)	0.0017 (6)	0.0080 (7)	−0.0005 (6)
C2	0.0412 (11)	0.0221 (8)	0.0390 (11)	0.0061 (8)	0.0132 (9)	−0.0005 (8)
C3	0.0290 (9)	0.0254 (8)	0.0314 (10)	−0.0022 (7)	0.0048 (8)	0.0023 (7)
C4	0.0303 (9)	0.0318 (9)	0.0358 (11)	0.0016 (7)	0.0113 (8)	0.0071 (8)
C5	0.0475 (14)	0.0690 (17)	0.0420 (14)	−0.0079 (12)	0.0134 (11)	0.0210 (12)
C6	0.0325 (14)	0.0377 (14)	0.0241 (13)	0.000	0.0115 (11)	0.000
C7	0.0279 (14)	0.074 (2)	0.0311 (16)	0.000	0.0051 (13)	0.000
C8	0.044 (2)	0.045 (6)	0.043 (2)	0.003 (2)	0.0020 (18)	0.001 (2)
C10	0.0330 (13)	0.0241 (11)	0.0263 (13)	0.000	0.0096 (11)	0.000
C11	0.0391 (17)	0.066 (2)	0.0323 (17)	0.000	0.0185 (14)	0.000
C12	0.059 (2)	0.091 (8)	0.0343 (19)	0.021 (7)	0.0253 (19)	−0.006 (6)
C13	0.070 (4)	0.148 (10)	0.061 (4)	−0.013 (4)	0.048 (3)	−0.011 (4)

### Geometric parameters (Å, º)

**Table:**

Sn—S1	2.5425 (5)	C6—C7	1.523 (4)
Sn—S2	2.9318 (5)	C6—H6A	0.9900
Sn—S1^i^	2.5425 (5)	C6—H6B	0.9900
Sn—S2^i^	2.9318 (5)	C7—C8	1.550 (5)
Sn—C6	2.146 (3)	C7—H7A	0.9900
Sn—C10	2.138 (3)	C7—H7B	0.9900
S1—C1	1.7443 (18)	C8—C9	1.498 (7)
S2—C1	1.6974 (19)	C8—H8A	0.9900
O1—C4	1.411 (2)	C8—H8B	0.9900
O1—C5	1.421 (3)	C9—H9A	0.9800
N1—C1	1.337 (2)	C9—H9B	0.9800
N1—C2	1.466 (2)	C9—H9C	0.9800
N1—C3	1.476 (2)	C10—C11	1.511 (4)
C2—H2A	0.9800	C10—H10A	0.9900
C2—H2B	0.9800	C10—H10B	0.9900
C2—H2C	0.9800	C11—C12	1.545 (5)
C3—C4	1.508 (3)	C11—H11A	0.9900
C3—H3A	0.9900	C11—H11B	0.9900
C3—H3B	0.9900	C12—C13	1.521 (8)
C4—H4A	0.9900	C12—H12A	0.9900
C4—H4B	0.9900	C12—H12B	0.9900
C5—H5A	0.9800	C13—H13A	0.9800
C5—H5B	0.9800	C13—H13B	0.9800
C5—H5C	0.9800	C13—H13C	0.9800
			
C10—Sn—C6	136.27 (11)	C7—C6—H6B	109.5
C10—Sn—S1	104.32 (6)	Sn—C6—H6B	109.5
C6—Sn—S1	107.55 (5)	H6A—C6—H6B	108.1
C10—Sn—S1^i^	104.32 (6)	C6—C7—C8	114.3 (3)
C6—Sn—S1^i^	107.55 (5)	C6—C7—H7A	108.7
S1—Sn—S1^i^	84.97 (2)	C8—C7—H7A	108.7
C10—Sn—S2	85.12 (2)	C6—C7—H7B	108.7
C6—Sn—S2	81.73 (2)	C8—C7—H7B	108.7
S1—Sn—S2	65.482 (14)	H7A—C7—H7B	107.6
S1^i^—Sn—S2	150.431 (15)	C9—C8—C7	107.9 (4)
C1—S1—Sn	93.17 (6)	C9—C8—H8A	110.1
C1—S2—Sn	81.45 (6)	C7—C8—H8A	110.1
C4—O1—C5	111.11 (19)	C9—C8—H8B	110.1
C1—N1—C2	120.35 (16)	C7—C8—H8B	110.1
C1—N1—C3	122.84 (15)	H8A—C8—H8B	108.4
C2—N1—C3	116.80 (15)	C8—C9—H9A	109.5
N1—C1—S2	121.49 (14)	C8—C9—H9B	109.5
N1—C1—S1	118.64 (14)	H9A—C9—H9B	109.5
S2—C1—S1	119.87 (10)	C8—C9—H9C	109.5
N1—C2—H2A	109.5	H9A—C9—H9C	109.5
N1—C2—H2B	109.5	H9B—C9—H9C	109.5
H2A—C2—H2B	109.5	C11—C10—Sn	114.6 (2)
N1—C2—H2C	109.5	C11—C10—H10A	108.6
H2A—C2—H2C	109.5	Sn—C10—H10A	108.6
H2B—C2—H2C	109.5	C11—C10—H10B	108.6
N1—C3—C4	113.55 (16)	Sn—C10—H10B	108.6
N1—C3—H3A	108.9	H10A—C10—H10B	107.6
C4—C3—H3A	108.9	C10—C11—C12	112.3 (3)
N1—C3—H3B	108.9	C10—C11—H11A	109.2
C4—C3—H3B	108.9	C12—C11—H11A	109.2
H3A—C3—H3B	107.7	C10—C11—H11B	109.2
O1—C4—C3	109.33 (17)	C12—C11—H11B	109.2
O1—C4—H4A	109.8	H11A—C11—H11B	107.9
C3—C4—H4A	109.8	C13—C12—C11	112.9 (5)
O1—C4—H4B	109.8	C13—C12—H12A	109.0
C3—C4—H4B	109.8	C11—C12—H12A	109.0
H4A—C4—H4B	108.3	C13—C12—H12B	109.0
O1—C5—H5A	109.5	C11—C12—H12B	109.0
O1—C5—H5B	109.5	H12A—C12—H12B	107.8
H5A—C5—H5B	109.5	C12—C13—H13A	109.5
O1—C5—H5C	109.5	C12—C13—H13B	109.5
H5A—C5—H5C	109.5	H13A—C13—H13B	109.5
H5B—C5—H5C	109.5	C12—C13—H13C	109.5
C7—C6—Sn	110.60 (19)	H13A—C13—H13C	109.5
C7—C6—H6A	109.5	H13B—C13—H13C	109.5
Sn—C6—H6A	109.5		
			
C2—N1—C1—S2	4.5 (3)	C1—N1—C3—C4	−91.6 (2)
C3—N1—C1—S2	−176.29 (14)	C2—N1—C3—C4	87.6 (2)
C2—N1—C1—S1	−175.26 (14)	C5—O1—C4—C3	−175.28 (18)
C3—N1—C1—S1	3.9 (2)	N1—C3—C4—O1	−62.2 (2)
Sn—S2—C1—N1	−178.28 (16)	Sn—C6—C7—C8	−170.1 (2)
Sn—S2—C1—S1	1.51 (10)	C6—C7—C8—C9	−76.4 (5)
Sn—S1—C1—N1	178.07 (14)	Sn—C10—C11—C12	−175.9 (11)
Sn—S1—C1—S2	−1.72 (11)	C10—C11—C12—C13	164.0 (11)

Symmetry code: (i) *x*, −*y*+1/2, *z*.

### Hydrogen-bond geometry (Å, º)

**Table:**

*D*—H···*A*	*D*—H	H···*A*	*D*···*A*	*D*—H···*A*
C4—H4*B*···S2^ii^	0.99	2.96	3.608 (2)	124

Symmetry code: (ii) *x*−1, *y*, *z*.
